# A deep cut into early cryptococcal pathogenesis

**DOI:** 10.1128/mbio.00657-24

**Published:** 2024-07-08

**Authors:** J. Muse Davis

**Affiliations:** 1 Stead Family Department of Pediatrics, Carver College of Medicine, University of Iowa, Iowa City, Iowa, USA; University of Florida College of Dentistry, Gainesville, Florida, USA

**Keywords:** *Cryptococcus*, pathogenesis, dissemination

## Abstract

Dissemination from one organ system to another is common to many pathogens and often the key process separating simple illness from fatal infection. The pathogenic *Cryptococcus* species offer a prime example. Cryptococcal infection is thought to begin in the lungs, as a mild or asymptomatic pneumonia. However, bloodborne dissemination from the lungs to the brain is responsible for the most devastating forms of infection. As with other disseminating infections, the transition likely depends on rare but crucial events, such as the crossing of a tissue barrier. By their nature, these events are difficult to study. Francis et al. (mBio 15:e03078-23, 2024, https://doi.org/10.1128/mbio.03078-23) have addressed this difficulty by developing a powerful imaging pipeline to scan through unprecedented volumes of tissue from mice infected with *Cryptococcus* at multiple stages of infection. Their observations challenge some of our basic assumptions about cryptococcal pathogenesis, including when and how the organism reaches the bloodstream and the central nervous system.

## COMMENTARY

Cryptococci are saprophytic fungi found in environmental niches such as pigeon guano (1). Prior to the advent of HIV, human cryptococcal infection was very rare and associated with other immunocompromising conditions (2). Cryptococcal meningoencephalitis is now a common initial diagnosis of full-blown AIDS (3), and our understanding of its pathogenesis has increased with the rising burden (1). It is generally thought that the fungus is inhaled into the deep airway and taken up by macrophages. It has a complex relationship with lung macrophages, and when unchecked by a functioning immune system, symptomatic disease in the lung ensues. From the lung, the fungus reaches the bloodstream and preferentially seeds the central nervous system (CNS).

Within this timeline of pathogenesis, some stages have been easier to study than others. Many forms of interactions with phagocytes have been described, and the fungus is capable of living and replicating inside these cells, exiting, re-entering, and transferring in between (4). The fate of *Cryptococcus* in the bloodstream has also been studied in mouse and zebrafish models (5, 6). *In vitro* models have been used to examine the direct and indirect interactions between *Cryptococcus* and brain endothelial cells (7), although these exclude many aspects of the complex structure separating the blood from the CNS. Finally, intrathecal infections have given access to cryptococcal growth in the CNS. Between the environment and the lung, the lung and the blood, and the blood and the CNS are missing links in our understanding that evade conventional approaches. Francis et al. have devised a high-throughput imaging pipeline to make interesting observations regarding these links.

In brief, the imaging pipeline involves infection of mice followed by euthanization and fixation of brain and lungs at specific timepoints. The tissues are then cut into thick sections for de-calcification and de-colorization, and examined via confocal microscopy (8). De-colorization of the tissues combined with automated microscopy allows for the assembly of high-resolution, three-dimensional image sets covering large portions of the cranium and lungs. These data sets are then examined manually or processed with automated software to quantify large areas. Combined with an array of immunochemical markers and dyes, this approach allows for unprecedented detail in the examination of murine cryptococcal infection.

Because human infection is thought to start with inhalation of the fungus, a common approach in the mouse has been to instill a substantial inoculum into the upper airway. Using high-volume microscopy of the nasal passages and the brain, the authors find that after this administration, a large amount of *Cryptococcus* remains in the deep nasal passages at 24 hours. They also find the yeasts there have already formed titan cells. Titan cells are unique cryptococcal cells, greater than 10 µm in diameter and highly resistant to phagocytosis (9). The ability to produce titan cells is an important factor in cryptococcal virulence. These cells are typically associated with well-established pulmonary infection, so it is surprising to find titan cell production induced anywhere so early in infection. The authors also find evidence suggesting that the nose itself can serve as the portal of entry, into the blood or perhaps directly into the brain. This mechanism has long been suspected in feline and canine cryptococcosis (10), although as yet there is scant evidence for it in humans.

The imaging pipeline used here also allows for observation of rare events very early after inoculation. They observed extracellular yeast in the pulmonary vasculature as early as 24 hours post infection. Had these arrived via the nasal tissues or the deeper lung? The authors also observed yeast in the brain at 7 days post infection, earlier than expected and confirming prior findings using whole-organ fungal cultures (11). These findings challenge the widely accepted timeline of cryptococcal pathogenesis in the mouse.

The emerging interpretation is that multiple mechanisms exist for cryptococcal transfer from the blood into the CNS (6, 12–16) and that a very small number of transfer events can account for florid CNS infection. The authors’ use of automated imaging of large volumes of tissue (large numbers of thick tissue sections) allows the examination and quantification of such rare and varied events. A long-standing question in cryptococcal pathogenesis is whether the fungus arrives at the CNS within phagocytes or if they interact directly with the endothelium themselves. At 3 and 7 days, the authors observed only extracellular fungi in the bloodstream, favoring the latter. It has been shown that *Cryptococcus* inoculated into the mouse bloodstream is rapidly removed from circulation (17), and direct live microscopy has shown instances of yeast passing through the brain endothelium in the hours after infection (6). Adding to these findings, the authors examined brain tissue at 24 hours post intravenous infection, finding yeast distributed throughout the brain, predominantly outside the vasculature and within or in contact with phagocytes expressing Iba1 (18). This marker is not only expressed by microglia, which reside in the parenchyma of the brain, but also by perivascular macrophages, which reside in a particular space between the brain endothelium and the limits of the parenchyma in arterioles and venules of the brain (19). A strong argument has been made for this perivascular space as a portal for cryptococcal dissemination (20).

Using a new high-throughput imaging approach, Francis et al. have made observations that challenge the commonly accepted narrative of cryptococcal pathogenesis. Rather than a linear path from the airway, to the lungs, to the bloodstream, and hence to the CNS, their findings suggest more rapid, alternative transit of yeast cells directly from the nasal cavity to the blood or brain. They also substantially support the proposition that *Cryptococcus* disseminates in the bloodstream alone but is rapidly associated with resident phagocytes in the CNS. These findings continue a fascinating but challenging pattern of unpredictability in cryptococcal pathogenesis: yeast and macrophages interact in a variety of ways, not just one, and with multiple outcomes. Yeast interact with brain endothelium in a variety of ways, with a common outcome of CNS dissemination. Perhaps the upper airway contributes to a variety of interactions and outcomes of its own.
